# EviSIP: using evidence to change practice through mentorship – an innovative experience for reproductive health in the Latin American and Caribbean regions

**DOI:** 10.1080/16549716.2020.1811482

**Published:** 2020-09-01

**Authors:** Suzanne J. Serruya, Rodolfo Gómez Ponce de León, Maria V. Bahamondes, Bremen De Mucio, Maria L. Costa, Pablo Durán, José L. Díaz-Rosello, Caron Kim, Antonella F. Lavelanet, Ana Artigas, Thais A. Forster, José G. Cecatti

**Affiliations:** a CLAP/WR-PAHO/WHO – Latin American Center of Perinatology/Women´s Health and Reproductive Health, Pan-American Health Organization, Montevideo, Uruguay; b University of Campinas School of Medicine and CEMICAMP (Center for Studies of Reproductive Health in Campinas), Campinas, Brazil; c Department of Sexual and Reproductive Health and Research, Preventing Unsafe Abortion Team. World Health Organization, Geneva, Switzerland

**Keywords:** Research method, mentorship, reproductive health, network, electronic health records, sip, perinatal information system

## Abstract

Maternal mortality is unacceptably high in our region. In 2015, the Latin American Center for Perinatology and Women´s Reproductive Health (CLAP) created a regional network of institutions including 16 countries, committed to improving epidemiological surveillance and healthcare of women in a situation of abortion or near miss event, using a common platform, the Perinatal Information System (SIP). The objective of the current pilot project was to test a new method of study called EviSIP (Evidence from SIP), a method of generating information on maternal near miss and abortion for the region. We describe the implementation of this initiative in reproductive healthcare facilities using SIP. Junior researchers/clinicians from these countries were included, along with expert researchers in reproductive health from across the world. Articles were produced with data on maternal near miss and abortion gathered from the SIP of each participating sentinel center; and recommendations from experts. EviSIP was the first joint workspace to discuss patient outcomes after treatment of abortion or near miss cases, with data analysis of each Sentinel Center; discuss and analyze data among centers, at a country and regional level; discuss the main outcomes and their impact on changing procedures and policies; strengthen the operational research capacity of the centers; develop and encourage the publication of scientific articles. The EviSIP initiative also promoted training of healthcare professionals in research. EviSIP provided a unique opportunity to train for research and mentorship and was pivotal to the production of scientific knowledge of reproductive health in the region.

## Background

Maternal mortality is unacceptably high. In 2017, about 295 000 women died worldwide, during and following pregnancy and childbirth. Most of these deaths (94%) occurred in low- and middle-income countries, and most could have been prevented with evidence-based interventions [1]. In addition to the tragic loss of life, maternal death can have negative effects on families. It can also affect the physical and mental health of family members. There is a great increase in mortality among children whose mothers died during or after their births. Other documented effects include catastrophic payments and reduced household income. Therefore, the risks of maternal deaths are not only elevated by poverty, but their occurrence may also perpetuate the cycle of poverty in poor communities from one generation to the next [2].

The first step in planning interventions and changing practices in healthcare in all settings is data sharing and analysis. Reliable and clear information is key to bringing awareness about priorities in maternal and perinatal health. Continuous data collection for prospective surveillance and local feedback is the goal and main difficulty in low -and-middle-income countries. The development of operational research can be challenging for national or regional networks [3,4]. Sustaining research networks, especially in the field of Sexual and Reproductive Health and Rights (SRHR) can be even more difficult, especially in terms of data sharing among different centers [5,6].

Over the last three decades, Latin America has had a unique opportunity to translate into practice the adequate use of a well-established data collection system implemented throughout countries. The purpose is to achieve a better quality of evidence-based care, which incorporates data on key topics, such as abortion and maternal near miss. Recognizing this opportunity, in 2015 the Latin American Center for Perinatology, Women and Reproductive Health (CLAP from now on) created a regional network of institutions committed to improving epidemiological surveillance and quality of care in women during pregnancy. To enter the network, centers should be appointed by health authorities of their countries. A sufficient number of cases; existence of computerized clinical records; availability of human resources; training systems and political willingness of hospital authorities were inclusion criteria for the network. This network – known as CLAP Network – has two components: NEAR MISS [7] and MUSA [8] (the Spanish acronym for women undergoing abortion). It includes 40 hospitals, known as Sentinel Centers (SC), scattered in 16 countries in the region [9]. These SC have been trained by CLAP to use electronic medical records based on the Perinatal Information System (SIP) [10], which includes a range of specific clinical records, for example cases of maternal near miss and women in a situation of abortion. It also allows the analysis, review and evaluation of data quality including evidence-based interventions required and recommended by WHO to improve women’s care. The aforementioned records were created following standard criteria proposed by the World Health Organization (WHO) with the support of the International Federation of Gynecology and Obstetrics (FIGO) [7].

SIP, a computerized clinical record system under technical support from CLAP is a landmark in the use of systematized information with several database options to explore for different purposes [10,11]. With a set of core indicators, all countries using the system are able to report some related health indicators derived from their dataset to their health authorities and the general public, receiving feedback from experts and/or authorities on outcomes and necessary interventions [12]. However, it is always important to consider that each setting, each country, and each health facility has its priorities and regional concerns.

Even with significant reductions in maternal and perinatal mortality in the last decade, major efforts are required to achieve the Sustainable Development Goals by 2030 in all Latin American and Caribbean countries [13,14]. Maternal and perinatal surveillance are needed to accelerate this progress, especially in countries with a high burden of maternal and perinatal morbidity and mortality. Research capacity should be strengthened to evaluate, translate and scale-up effective interventions that will result in changes in health policies. Individuals participating in a data collection network must have the space to ascertain the quality of information generated and/or received [6].

Based on difficulties in publishing and reporting data on maternal morbidity, near miss and abortion, encountered by several Latin American health facilities, CLAP decided to initiate a new activity with the affiliated SC.

In one of the most comprehensive systematic reviews published in 2014, Gagliardi et al. define that successful formal mentoring programs that are meant to enhance knowledge, skills, or performance, may include individual and/or group mentoring offered by a senior or expert mentor. After reviewing 13 eligible studies, those authors concluded that mentoring programs that are likely to form the basis of Knowledge Translation mentorship have the following key components: a combination of preliminary workshop-based training and individual mentoring, provided either in person or remotely; training of mentors; and periodic mentoring for at least an hour over a minimum period of six months [15].

Mentorship is known to be a key feature of research capacity development. However, the resources that enable such a practice, organizational impediments and lack of standard guidance are main limitations to the empowerment of new investigators and partnerships. A focus on south-to-south collaboration, with the support of renowned regional leaders augments the mentoring initiative and shifts towards improving the quality of maternal and perinatal healthcare.

## Methods

### CLAP Network (the concept)

In 2015, in the introductory sessions of the first Network meetings in Brasilia [13] and Panama [16], CLAP’s Director said: ‘Maternal mortality has been reduced considerably in the last 20 years, but it continues to be unacceptably high, and the majority of its causes can be prevented or treated’. On the same occasion, it was proposed that ‘The new CLAP Network will play an important role in helping to further reduce maternal and neonatal mortality in the Region by collecting data on maternal and neonatal death causes, in addition to causes of complications that can seriously affect women following childbirth’ [16].

At the next meeting held in Bogota, Colombia in 2017 [9], it was established that one of the Network’s key purposes was to work on three elements related to improved use of SIP data and resources:

1. Data Review: Create protocols and timetables to ensure that the information collected at the centers is well captured, and work on the appropriation of data completion exercise by the healthcare staff at each center;
2. Use of information for Health System management: Through good data collection and analysis, contribute to the creation and transformation of strategies that promote quality and timely healthcare of pregnant women or women considering/requesting induced abortion in the Health System.
3. Use of information for operational research: identify SC to develop operational research in the region, thus providing information to improve health care.

SCs receive ongoing support from an external institution (UNICEM Clinical & Epidemiological Research Unit Montevideo [17]) in charge of data management coordination, data quality control and continuous quality of care monitoring. UNICEM sends monthly reports to SC, with alerts highlighting inconsistencies and gaps to be solved in their databases. It also provides a report on quality of care assessment and ranking of the center based on regional quality indicators. The monthly report on quality of care typically contains information on maternal morbidity status, use of procedures or drugs for pain relief, use of antibiotic prophylaxis, and use of evidence-based clinical practice following WHO recommendations, among others.

Following network implementation, CLAP launched an innovative approach to mentorship in 2019, using methodologies applied in business and administration. These meetings were conducted in a protected environment, with adequate time allocation. For this initiative, such an event was called: ‘EviSIP: using evidence for decision making’. This event was not only a workshop, conference, or meeting or training section, but rather it was the culmination of six weeks at a distance mentorship process, followed by a four-day face-to-face immersion in mentoring, as well as intensive data analysis and writing. Representatives of selected centers from 10 countries shared their databases on maternal near miss and/or abortion and addressed their relevant questions in the meeting. Participants learned to use information stored on the database and plan, present and analyze their data to produce a manuscript with valuable knowledge. By the end of the week, all groups had submitted a manuscript draft. The manuscript is the first result we can measure, although the intended product goes far beyond publication.

Therefore, the aim of this manuscript is to detail the concept, processes, methods, procedures and results of this innovative experience to support future mentorship, especially among people and institutions from low- and middle-income settings in the analysis of local healthcare information, to improve sexual and reproductive healthcare in women.

### Small grant proposals (the process)

**Figure 1.**
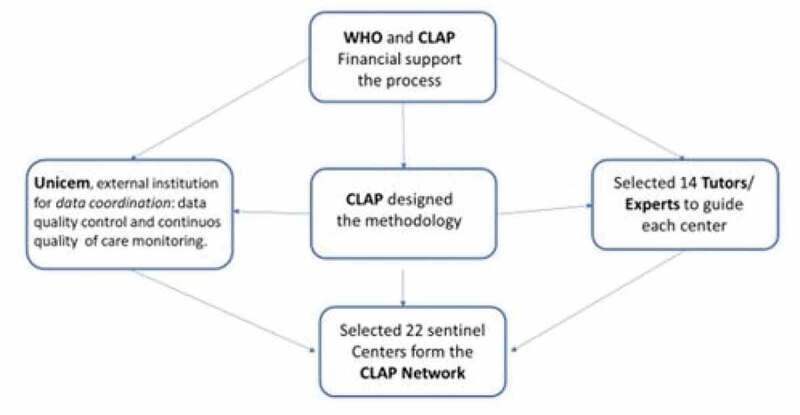
Flow chart steps done to recruit and build the EviSIP meeting.

In June 2019, CLAP issued a call to the network´s SC for research proposals using data collected on SIP database (Figure 1). The purpose of this call was to create a space where SC with the most consistent research proposals would be able to work with international experts and their peers in research product development and analysis of their own data.

Selected healthcare professionals from each sentinel center at different stages of their careers that have regularly participated in webinars every month since 2017, reviewing quality of care and updating clinically relevant evidence, were invited to intensify the frequency of these activities, in preparation for the in-person meeting. These professionals participated in one-hour once-weekly online meetings, where they received new information on research methods related to different areas of interest. Special focus was on Operational Research, using WHO guidelines on quality of care standards. Meetings included information on how to write a scientific manuscript [18], review bibliography [19] and other research priorities [20,21]. They also had the opportunity to improve their research training with a two-month online course offered by the Latin American WHO-HRP hub – the Center for Studies in Reproductive Health of Campinas (Cemicamp) [22] under the initiative of HRP (Human Reproduction Programme) Alliance.

**Figure 2.**
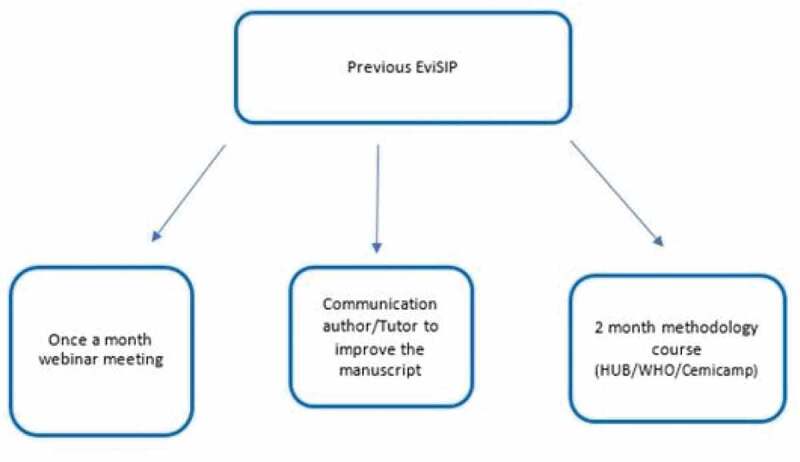
Flow chart steps done previously EviSIP meeting.

A step-by-step explanation was later given during an online meeting (Figure 2). Countries were informed about how the small grants project would work, what was expected from them, and how to achieve those goals. Funds could only be used for recruitment of people to reduce missing data and inconsistencies in the SIP database and informatics equipment for SIP implementation. Only four out of 37 health care providers participating in the EviSIP experience had previous publications in co-authorship in peer-reviewed journals indexed in PubMed or SCiELO. All were certified clinicians.

### Mentorship (the process)

Fourteen mentors from different areas of expertise including statistics, epidemiology, maternal and child health, and ethics were assigned to work with the SC and their researchers. Mentors were chosen based on their field of interest and expertise, previous experience in dealing with research methods in the PAHO and WHO context, and some language skills in English and Spanish. The latter was predominantly spoken by the majority of attendees. Each mentor was assigned to a center according to their field of expertise and selected topic to be developed in their projects.

During the six weeks before the meeting, mentors and mentees had the opportunity to engage in virtual meetings and exchange emails to discuss the plan of analysis, drafts or manuscripts and implement changes, based on information from a data cleaning process, management, or analysis implemented. The extensive data cleaning plan consisted of:

- External quality control team (UNICEM) mapping inconsistencies, providing feedback and requesting corrected bases. This step was repeated on a weekly/fortnightly basis, according to the demand of the SC and based on their capacity to respond. SC started to correct their databases for analysis during the in-person meeting.
- SC received their databases to start analysis from one to two months before the meeting, depending on how the level of corrections progressed. The last databases were delivered on the same day as the meeting started.
- UNICEM team participated before and during the workshop, providing technical support and collaborating with their analysis.

The SCs presented research proposals using data from the SIP database, either on abortion or near miss. Afterwards, their initial analysis and screening had to be completed within 15 days. Candidates were told that the process would end with the presentation of the operational research at the Network’s face-to-face meeting. Twenty-five proposals were received, 24 of which were approved. The meeting gathered 22 SCs from 10 Latin American countries (Argentina, Bolivia, Brazil, Chile, Colombia, Cuba, Dominican Republic, Guatemala, Honduras, and Nicaragua), 18 from the MUSA Network and six from the NEAR MISS Network. Two SC provided proposals for both Networks, one from Colombia and another from Honduras.

Regarding the method used in the projects, the cross-sectional study was the most commonly chosen design. Only one center proposed a case-control study. The mentors considered the selected design the most appropriate way to describe local problems, show results in their countries, and propose institutional changes. In all cases, the authors defined the analysis criteria more clearly after receiving feedback.

Among the 24 projects assessed, 10 had improved their methods (with better-designed study objectives and analysis plans). Through mentor advice and webinar meetings, eight improved their bibliographic search. Before the meeting, three out of 22 centers presented a consolidated and complete project.

## Results: the EviSIP meeting

A four-day meeting took place in Montevideo, Uruguay. At the very beginning, there was a socializing activity so that mentees and tutors could meet face-to-face. Subsequently, there were different spaces for lectures, writing scientific articles and parallel meetings among mentors.

During the first day, meetings were held by local authorities, CLAP, PAHO and WHO representatives. Mentees had time to discuss work plans with mentors to finalize protocol improvements (this space was called ‘alignment session with mentors’). Finally, the authors had a space to hold their first writing session and put into practice the recommendations received.

**Figure 3.**
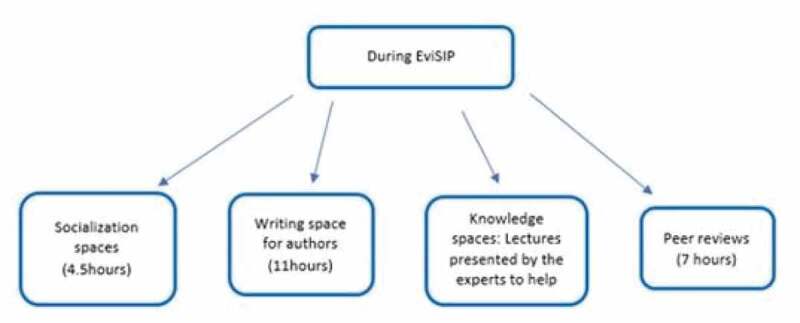
Flow chart steps done during EviSIP meeting.

On the second day, some mentors gave lectures on topics related to methods and quality writing of a scientific article. Then, there was another ‘alignment session with mentors’, followed by a second writing session for mentees. Meanwhile, a parallel meeting of the mentors was proposed to discuss and define recommendations for a collaborative writing method in the region for SRH (Figure 3). A methodology termed ‘in-person blind peer review’ was also conducted, in which each mentor was assigned one or two projects previously unknown to them. Then, mentees received anonymous comments, criticisms and/or suggestions about their projects, simulating the process of submitting an article to a scientific journal and receiving comments from the reviewers. This was an opportunity to practice a proper response to the recommendations suggested by the editor and peer review.

After the first peer review, according to information contained on a structured form provided by the reviewers, there was a suggestion to improve or prepare the abstracts in 80% of the projects. It was also suggested that the tables and references should be improved in 80% and 55% of the projects, respectively. Following the second peer review, 60% of the centers improved the abstracts, 70% had improved the tables, and 50% had improved the references. Thirty per cent (30%) of these centers improved only one of the suggested points, and two out of 22 made a minor improvement after two peer-review cycles. Regarding the topic studied, the vast majority of the subjects was considered important, relevant and adequate for scientific publication.

The experts invited to participate in the meeting had an open space for dialogue and discussion about some key recommendations for the development of ideas. The purpose was to enhance and promote capacity building, to obtain the best use of data for decision-making, improving the quality of care in pregnant women and women experiencing abortions (Supplementary file S1).

Briefly, the content of recommendations made by the experts arising from their specific discussions covered the following questions: what for, why, what, how and for who is epidemiological surveillance and use of reproductive health care systematically collected; development of surveillance newsletters and management of a situation room; research recommendations and use of data systematically for research; development of research products; capacity building and training; creation of a minimum core team; and finally, data applicability in public policy to promote health policy dialogues.

By the end of the meeting, all groups improved and completed their proposals. Six manuscripts were already sent to a peer-reviewed journal, 11 were in final editing and five were still in the writing phase. The CLAP staff and some international experts participating in the initiative are still committed to supporting those researchers so that their good manuscript can be accepted for publication.

## Discussion

The iterative process of proposal development and feedback from the SC and mentors strengthened the scientific content of the projects, with considerable improvement in the hypotheses and proposed data analyses. The EviSIP exercise has shown that the SC teams need to work on technical capacity-building, to improve their work and data collection in a strategic and scientific manner. The data collected can be used as local evidence, in addition to input to feed national and regional health-related informed decision-making and improve the conditions, healthcare and services provided.

The analysis of this information, the active spaces for data discussion, the creation of expert groups, continuing education and opportunities to discuss health public policies were considered pivotal to the process by participants. These may be positive steps to promote the effective use of routine data collected at the facilities, improving the quality and proper timing of women’s care.

The activity of mentorship, especially south-to-south is the key to increase awareness about the main topics of women´s health. The intention of having senior mentors together to discuss recommendations on tutoring and research was to shed light on pathways that make data operational, improve quality of care and increase the effectiveness and accuracy of health policies. These activities among mentors generated a brainstorm session on priorities regarding mentorship in low- and middle-income countries (Supplementary file S1). This is one of the strongest points of the initiative, given that these recommendations are not exclusive for this set of centers, but also applicable to other centers worldwide that may deal with different topics.

According to Gagliardi, the mentoring model may require one or more mentors with differing views and experience or those who can address multiple needs over time. Therefore, further research could explore whether coaching, mentoring or both are best for the development of knowledge translation capacity [15].

CLAP developed an innovative approach to mentorship and learning from one another through the network. Based on effective methods used in industry including bringing together teams that work under protected conditions (time and space), research projects in such mentoring methods had a few limitations: 1. time was restricted for the SC to write and submit projects, 2. participants had a diverse background and experience, 3. participants had limited time to work with mentors before their face-to-face meeting. However, this initiative also has several strengths, including an innovative approach to conduct data analysis, active discussion about the center’s own data, the creation of expert groups with continuing education and public health policy discussion. This process can be a positive step in promoting the effective use of data to improve quality of care.

## Conclusion

EviSIP provided a great opportunity for methodological training and mentorship since most centers did not have previous training in research. Through this process, the MUSA and NEAR MISS Networks allowed local and regional clinicians to expand their knowledge and skillset in the field of research. As a result, this process not only built local capacity, especially in post-abortion and postpartum care but it also provided an opportunity to improve surveillance and create and impact on quality of care.

## Paper context

There are no reported initiatives facilitating the process of preparing scientific proposals based on clinical activities. This is the first initiative designed to stimulate health professionals with training in research to produce knowledge of reproductive health in the Latin American and Caribbean regions. The joint effort and support of several institutions and expert professionals in the field plus the provision of small grants for the task were pivotal to the performance of proposed activities.
